# Supplementary material for: RAG suppresses group 2 innate lymphoid cells
Source: bioRxiv. 2025 Mar 20:2024.04.23.590767. Originally published 2024 Apr 28. Preprint. [Version 2] doi: 10.1101/2024.04.23.590767 (PMC11071423; doi:10.1101/2024.04.23.590767)

bioRxiv preprint doi: https://doi.org/10.1101/2024.04.23.590767; this version posted March 20, 2025. The copyright holder for this preprint (which was not certified by peer review) is the author/funder, who has granted bioRxiv a license to display the preprint in perpetuity. It is made available under aCC-BY 4.0 International license.

Figure S1. ILC2 and IL-5/IL-13 gating.

Gating for **A)** CD45$^+$, CD90$^+$, Lin$^-$ cells (Lin$^-$ defined as CD3$^-$, CD5$^-$, CD11b$^-$, CD11c$^-$, CD19$^-$, NK1.1$^-$, and FcεR1$^-$), then gating on **B)** ILC2 (IL-33R$^+$ Lin$^-$) corresponding to **Fig. 1C**, with subsequent gating of **C)** IL-5$^+$ and IL-13$^+$ ILC2, corresponding to **Fig. 1D-E.**

bioRxiv preprint doi: https://doi.org/10.1101/2024.04.23.590767; this version posted March 20, 2025. The copyright holder for this preprint (which was not certified by peer review) is the author/funder, who has granted bioRxiv a license to display the preprint in perpetuity. It is made available under aCC-BY 4.0 International license.

Figure S2. Expansion and activation of ILC2s in RAG2 deficiency compared to littermates. **A)** Schematic of steady state analysis of WT B6 (Control) mice or *Rag2*$^{-/-}$ mice. **B)** Proportion of CD90^{+} Lin^{-} cells (Lin- defined as CD3^{-}, CD5^{-}, CD11b^{-}, CD11c^{-}, CD19^{-}, NK1.1^{-}, and FcεR1^{-}) determined to be ILC2s (IL-33R^{+}) in sdLN at steady state from WT or *Rag2*$^{-/-}$ mice. Percent ILC2 from sdLN at steady state following PMA/iono stimulation positive for **C)** IL-5 or **D)** IL-13 staining. Data representative of 2 independent experiments, 2-3 mice per group. * P <0.05, ** P < 0.01 by two-tailed Welch's t test. All data represented as mean with standard deviation.

bioRxiv preprint doi: https://doi.org/10.1101/2024.04.23.590767; this version posted March 20, 2025. The copyright holder for this preprint (which was not certified by peer review) is the author/funder, who has granted bioRxiv a license to display the preprint in perpetuity. It is made available under aCC-BY 4.0 International license.

Figure S3. Confirmation of splenocyte reconstitution in splenocyte chimera mice.

Proportion of CD45$^+$ splenocytes from splenocyte chimera mice (related to **Fig. 2A-E**) determined to be **A)** CD4$^+$ T cells (CD4$^+$, CD8$^-$, CD19$^-$), **B)** CD8$^+$ T cells (CD4$^-$, CD8$^+$, CD19$^-$), **C)** B cells (CD4$^-$, CD8$^-$, CD19$^+$), and **D)** Eosinophils (SiglecF$^+$, CD4$^-$, CD8$^-$).

Figure S4. Donor cell reconstitution and gating in sdLN of WT:*Rag1*$^{-/-}$ bone marrow chimera mice.

A) Gating of live host (CD45.1$^+$) and donor (CD45.2$^+$) cells then **B)** Gating on donor cells by genotype (CD90.1$^+$ = WT [blue]; CD90.2$^+$ = Rag1$^{-/-}$ [orange]) in Lin$^-$ population then **C**) Gating on Lin$^-$ IL-33R$^+$ ILC2s in the skin draining lymph node (sdLN). **D**) Host/donor CD45$^+$ cell reconstitution in sdLN of WT:*Rag1*$^{-/-}$ bone marrow chimera mice. **E)** quantification of CD45.2$^+$ Lin$^-$ donor cells by genotype in sdLN of WT:*Rag1*$^{-/-}$ bone marrow chimera mice. **F**) total numbers of ILC2s normalized to 10^5 live cells and **G**) ILC2 proportion of Lin- cells in the sdLN. **H**) Gating for IL-5 and IL-13 after in vitro stimulation and intracellular cytokine staining of ILC2s from sdLN. Quantification of total positive cells normalized to 10^5 live cells for **I**) IL-5 and **J**) IL-13 and proportion of ILC2 positive for **K**) IL-5 and **L**) IL-13. **E,G,I-L:** * $P < 0.05$, ** $P < 0.01$ by ratio means paired t test. **D,F**: ** $P < 0.01$, *** $P < 0.001$, **** $P < 0.0001$, by RM one-way ANOVA test with Geisser-Greenhouse correction. All data represented as mean with standard deviation. Related to **Fig. 2G-J.**

bioRxiv preprint doi: https://doi.org/10.1101/2024.04.23.590767; this version posted March 20, 2025. The copyright holder for this preprint (which was not certified by peer review) is the author/funder, who has granted bioRxiv a license to display the preprint in perpetuity. It is made available under aCC-BY 4.0 International license.

Figure S5. sdLN multiome experiment.

A) *Rag1*^{Cre}::*Rosa26*$^{LSL\text{-}tdRFP}$ reporter mice were given topical treatments with 2 nmol MC903 dissolved in ethanol vehicle or with ethanol vehicle alone to each ear daily for 7 days. Harvested sdLN processed using Magnetic Activated Cell Sorting (MACS) led to depletion of cells expressing the CD3, CD19, and CD11b lineage markers and remaining cells were further processed in the 10X Multiome pipeline, generating both single cell RNA-sequencing and single cell ATAC-sequencing data for each cell. **B)** Ear thickness measured daily in the AD-like disease multiome experiment. Data representative of one experiment, with 4 mice per group pooled for sequencing. **** P <0.0001 by 2-way ANOVA with Sidak's multiple comparisons test, day 7. All data represented as mean with standard deviation.

bioRxiv preprint doi: https://doi.org/10.1101/2024.04.23.590767; this version posted March 20, 2025. The copyright holder for this preprint (which was not certified by peer review) is the author/funder, who has granted bioRxiv a license to display the preprint in perpetuity. It is made available under aCC-BY 4.0 International license.

Figure S6. ILC2 marker genes identified in the differentially accessible open chromatin assay.

Differentially accessible (DA) open chromatin peaks identified for the ILC2 cluster are highlighted in gray and shown next to the closest gene for **A)** Neuromedin U receptor 1 (*Nmur1*) and **B)** IL-5 (*Il5*). See **Table S3** for top 100 DA peaks and distances to nearest genes for the ILC2 cluster.

Figure S7. Dotplots of selected ILC2 marker genes.

Dotplots comparing selected marker genes from the multiomic ILC2 gene set (**Figure 4I, Table S4**) for each cluster between the GEX and GA assays, with genes highlighted by color corresponding to individual assays or overlap of assays in which they were identified. *Rora* was not detected in the GA assay.

bioRxiv preprint doi: https://doi.org/10.1101/2024.04.23.590767; this version posted March 20, 2025. The copyright holder for this preprint (which was not certified by peer review) is the author/funder, who has granted bioRxiv a license to display the preprint in perpetuity. It is made available under aCC-BY 4.0 International license.

Figure S8. Gene set enrichment analysis of differentially expressed genes in ILC2s.

A) Volcano plot of differentially expressed genes (DEGs) by RAG fate map for the ILC2 cluster. A ranked list (**Table S5**) was constructed for all DEGs with $\log_2$(fold change) >0.1 for gene set enrichment analysis (GSEA). **B)** Dotplot of GSEA result calculated using ClusterProfiler and the gene ontology (GO) biological process (BP) database (see methods). Full results in **Table S6**. **C)** GSEA plot of the GO BP “positive regulation of immune system process” gene set. $RAG^{naïve}$, RAG fate map negative; RAG^{exp}, RAG fate map positive.

Figure S9. Mapping gene to peak links in select ILC2 genes.

Gene to peak links (GPLs) mapped for the $RAG^{naïve}$ and RAG^{exp} states as depicted in **Figure 5B** for **A)** GATA binding protein 3 (*Gata3*) and **B)** Nedd4 family interacting protein 1 (*Ndfip1*). Only GPLs that fit in the coverage window are shown. Select peaks (teal bars) present in one state, but not the other, are highlighted in teal boxes. Full gene names not shown on figure in (A) are **9230102O04Rik* and ***4930412O13Rik* and in (B) #*Gm42690*.

bioRxiv preprint doi: https://doi.org/10.1101/2024.04.23.590767; this version posted March 20, 2025. The copyright holder for this preprint (which was not certified by peer review) is the author/funder, who has granted bioRxiv a license to display the preprint in perpetuity. It is made available under aCC-BY 4.0 International license.

Figure S10. Multiomic transcription factor analysis of ILC2s

A) Schematic of assigning transcription factor (TF) motif enrichment in differentially open chromatin to cell clusters using chromVAR. **B**) Heatmap of top 5 TF motif activity scores for each cluster. **C**) Dotplots comparing expression levels of selected TFs in the gene expression (GEX) assay with the chromVAR activity score of the corresponding TF motif. The TFs and corresponding motifs for *Rora* and *Rorg* in the ILC2 cluster are highlighted by boxes. An expanded list of cluster TF motif markers identified using chromVAR is in **Table S10**. **D**) Analysis of TF motifs enriched in ILC2 gene to peak links (GPLs) unique to $RAG^{naïve}$ and RAG^{exp} populations determined using the FindMotifs function in Signac. The top 6 TF motifs for each population are shown and are ranked by the $-log_{10}$ transformed false discovery rate (FDR - Bonferroni corrected p values). An expanded list is in **Table S11**. **E**) Dotplot of gene expression for TFs corresponding to the top enriched TF motifs identified in ILC2 GPLs from (D). TF genes that were not detected in the GEX assay are labeled N.D.

bioRxiv preprint doi: https://doi.org/10.1101/2024.04.23.590767; this version posted March 20, 2025. The copyright holder for this preprint (which was not certified by peer review) is the author/funder, who has granted bioRxiv a license to display the preprint in perpetuity. It is made available under aCC-BY 4.0 International license.

Figure S11. Gene to peak link analysis by RAG fate map and disease for all detected genes. **A)** UpSet plot of overlaps in peaks identified from GPLs of all genes split by both RAG fate map (RAG^{exp} and $RAG^{naïve}$) and disease state (SS - steady state, AD - AD-like inflammation). Each row corresponds to one of the four sets, and each column corresponds to an intersection of one or more sets (see methods). See **Table S13** for full list of peaks from GPLs for all genes in each set. Columns identifying key intersections are color coded by the corresponding RAG fate map or disease groups. The blue column indicates the intersection of peaks from $RAG^{naïve}$ cells and peaks induced by AD-like disease in RAG^{exp} cells.

bioRxiv preprint doi: https://doi.org/10.1101/2024.04.23.590767; this version posted March 20, 2025. The copyright holder for this preprint (which was not certified by peer review) is the author/funder, who has granted bioRxiv a license to display the preprint in perpetuity. It is made available under aCC-BY 4.0 International license.

Figure S12. Multiomic transcription factor analysis of Th2 locus

A) Analysis of TF motifs enriched in Th2 locus subset of ILC2 gene to peak links (GPLs) unique to $RAG^{naïve}$ and RAG^{exp} cell populations determined using the FindMotifs function in Signac. The top 6 TF motifs for each population are shown and are ranked by the $-log_{10}$ transformed false discovery rate (FDR - Bonferroni corrected p values). No TFs met the -log10(FDR) minimum cutoff value of 1.5 in the RAG^{exp} cell population. The full list of enriched motifs is in **Table S16**. **B**) Dotplot of gene expression for TFs corresponding to the top enriched TF motifs identified in Th2 locus GPLs from (A). TF genes that were not detected in the GEX assay are labeled N.D.

Figure S1

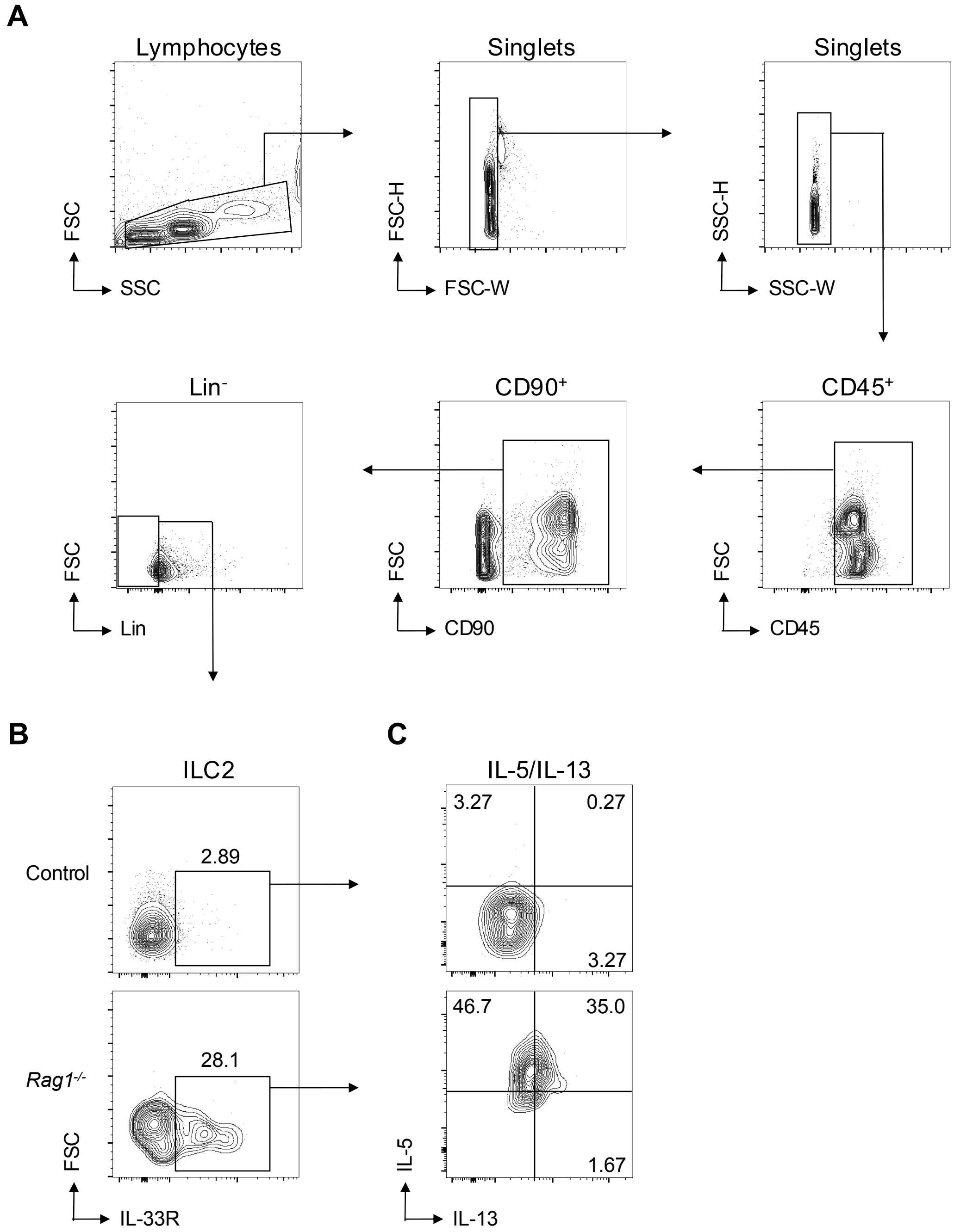
A
Lymphocytes
Singlets
Singlets
FSC
SSC
FSC-H
FSC-W
SSC-H
SSC-W
Lin-
CD90+
CD45+
FSC
Lin
FSC
CD90
FSC
CD45
B
C
ILC2
IL-5/IL-13
Control
2.89
3.27
0.27
3.27
Rag1-/-
28.1
46.7
35.0
1.67
FSC
IL-33R
IL-5
IL-13

Figure S2

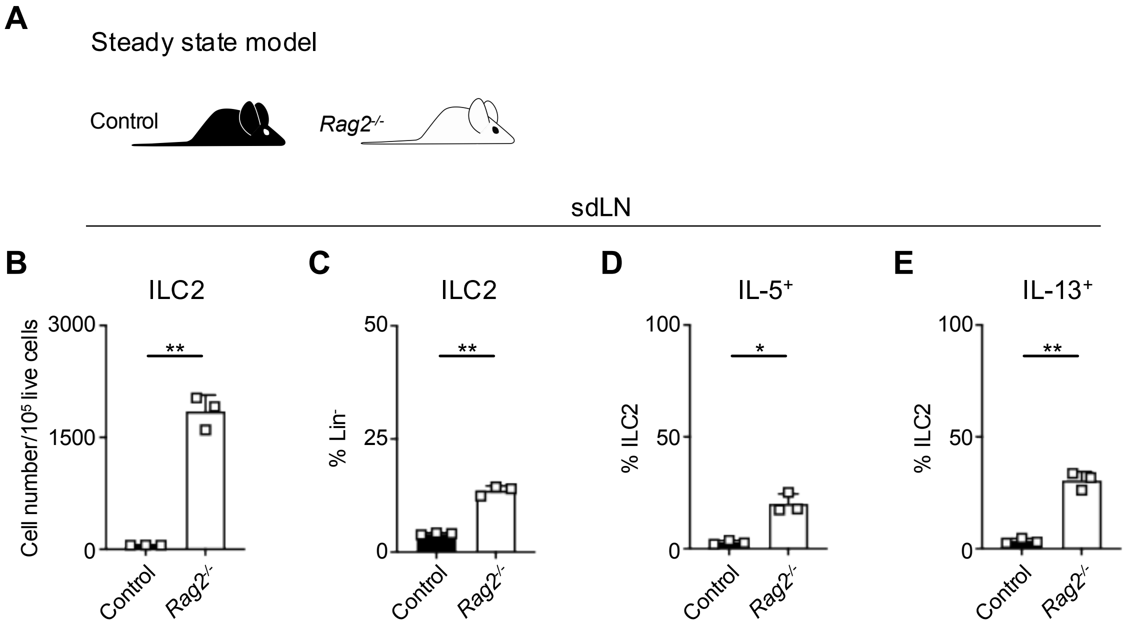
A
Steady state model
Control
Rag2-/-
sdLN
B
ILC2
Cell number/10⁵ live cells
3000
1500
0
**
Control
Rag2-/-
C
ILC2
% Lin-
50
25
0
**
Control
Rag2-/-
D
IL-5+
% ILC2
100
50
0
*
Control
Rag2-/-
E
IL-13+
% ILC2
100
50
0
**
Control
Rag2-/-

Figure S3

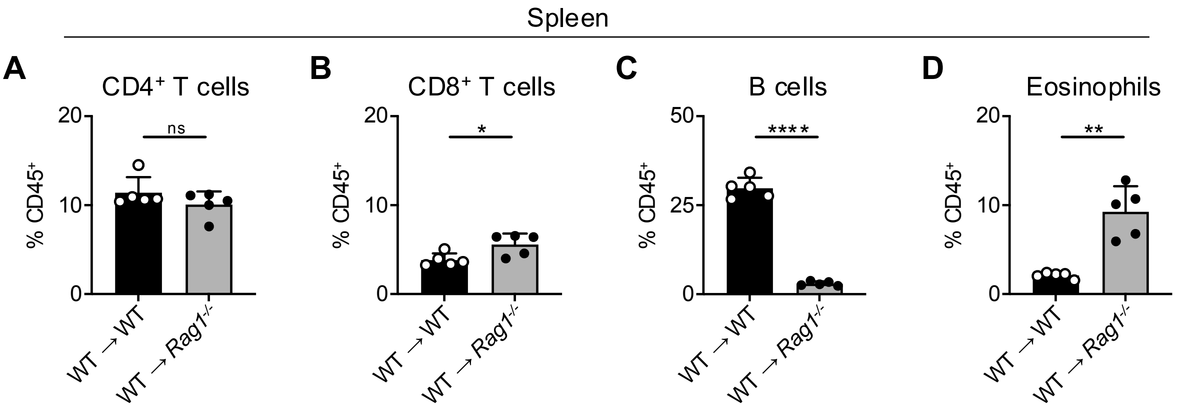

Spleen
A
CD4⁺ T cells
20
10
0
% CD45⁺
ns
WT → WT
WT → Rag1-/-
B
CD8⁺ T cells
20
10
0
% CD45⁺
*
WT → WT
WT → Rag1-/-
C
B cells
50
25
0
% CD45⁺
****
WT → WT
WT → Rag1-/-
D
Eosinophils
20
10
0
% CD45⁺
**
WT → WT
WT → Rag1-/-

Figure S4

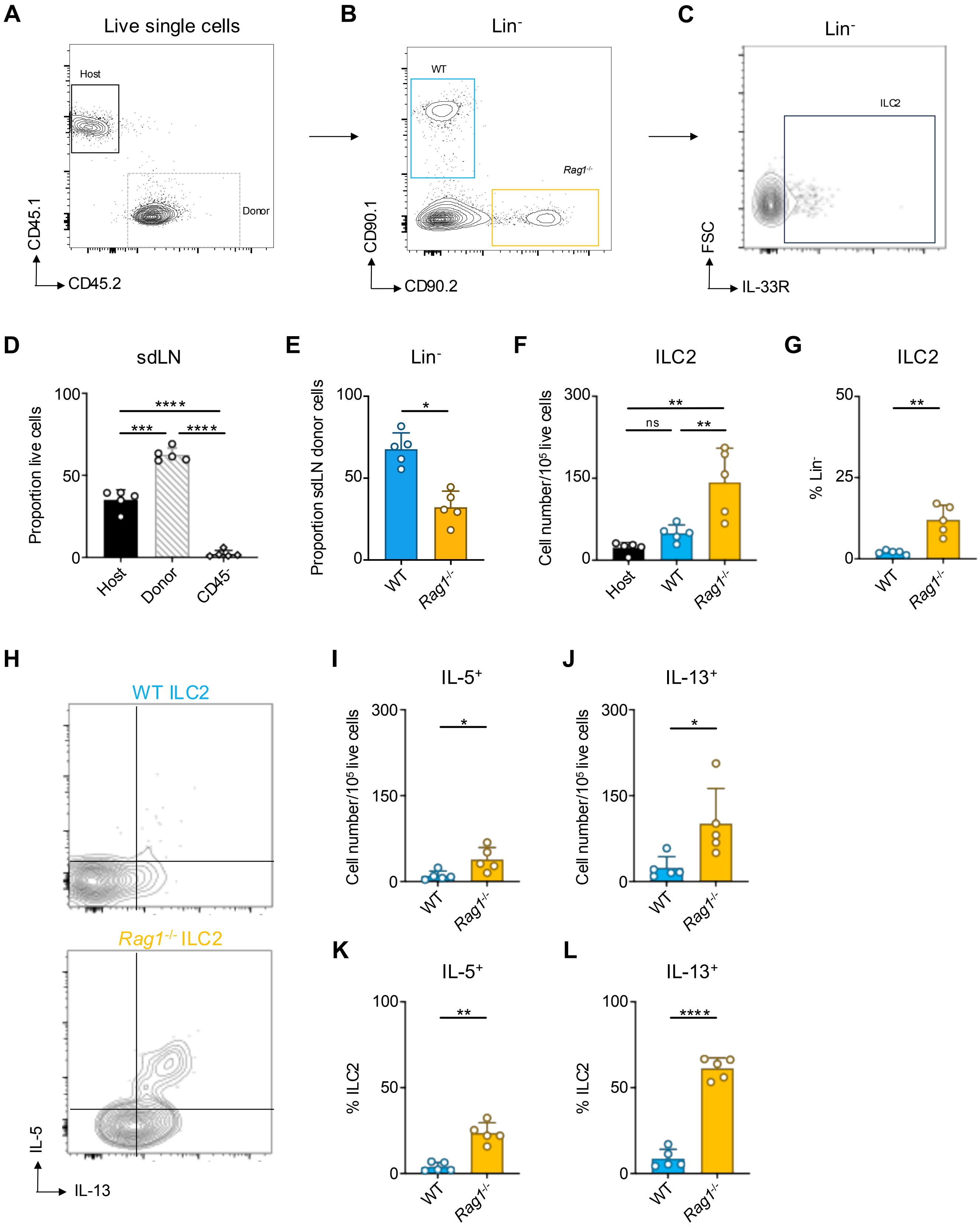
A
Live single cells
Host
Donor
CD45.1
CD45.2
B
Lin-
WT
Rag1-/-
CD90.1
CD90.2
C
Lin-
ILC2
FSC
IL-33R
D
sdLN
Proportion live cells
100
50
0
****
***
****
Host
Donor
CD45-
E
Lin-
Proportion sdLN donor cells
100
50
0
*
WT
Rag1-/-
F
ILC2
Cell number/10^5 live cells
300
150
0
**
ns
**
Host
WT
Rag1-/-
G
ILC2
% Lin-
50
25
0
**
WT
Rag1-/-
H
WT ILC2
Rag1-/- ILC2
IL-5
IL-13
I
IL-5+
Cell number/10^5 live cells
300
150
0
*
WT
Rag1-/-
J
IL-13+
Cell number/10^5 live cells
300
150
0
*
WT
Rag1-/-
K
IL-5+
% ILC2
100
50
0
**
WT
Rag1-/-
L
IL-13+
% ILC2
100
50
0
****
WT
Rag1-/-

Figure S5

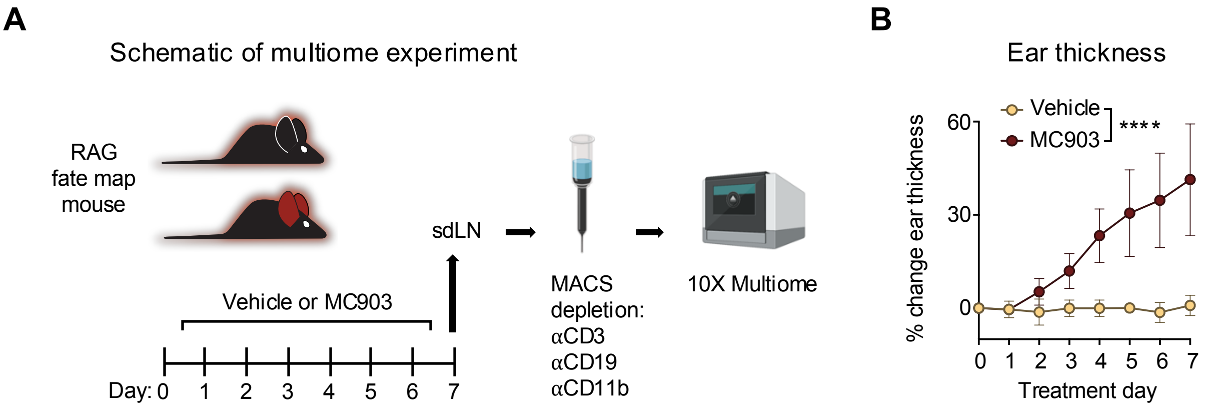
A
Schematic of multiome experiment
RAG
fate map
mouse
sdLN
MACS
depletion:
αCD3
αCD19
αCD11b
10X Multiome
Vehicle or MC903
Day: 0 1 2 3 4 5 6 7
B
Ear thickness
Vehicle
MC903
****
% change ear thickness
60
30
0
0 1 2 3 4 5 6 7
Treatment day

Figure S6

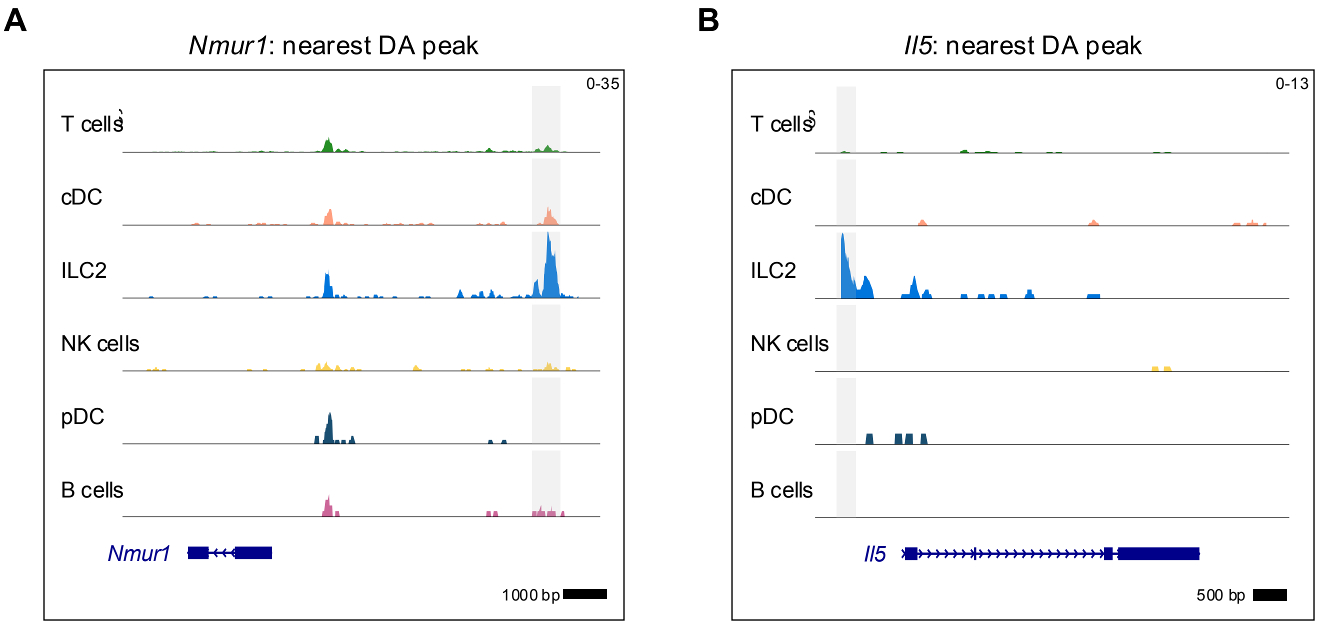
A
Nmur1: nearest DA peak
0-35
T cells
cDC
ILC2
NK cells
pDC
B cells
Nmur1
1000 bp
B
Il5: nearest DA peak
0-13
T cells
cDC
ILC2
NK cells
pDC
B cells
Il5
500 bp

Figure S7

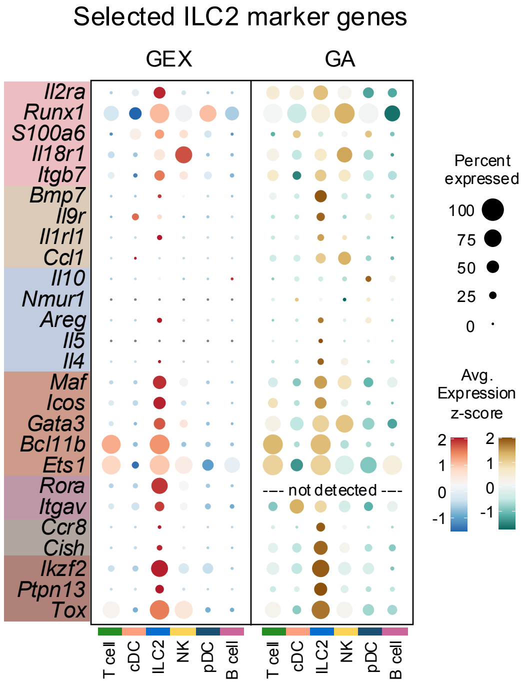

Selected ILC2 marker genes
GEX
GA
Il2ra
Runx1
S100a6
Il18r1
Itgb7
Bmp7
Il9r
Il1rl1
Ccl1
Il10
Nmur1
Areg
Il5
Il4
Maf
Icos
Gata3
Bcl11b
Ets1
Rora
Itgav
Ccr8
Cish
Ikzf2
Ptpn13
Tox
--- not detected ---
T cell
cDC
ILC2
NK
pDC
B cell
Percent expressed
100
75
50
25
0
Avg. Expression z-score
2
1
0
-1

Figure S8

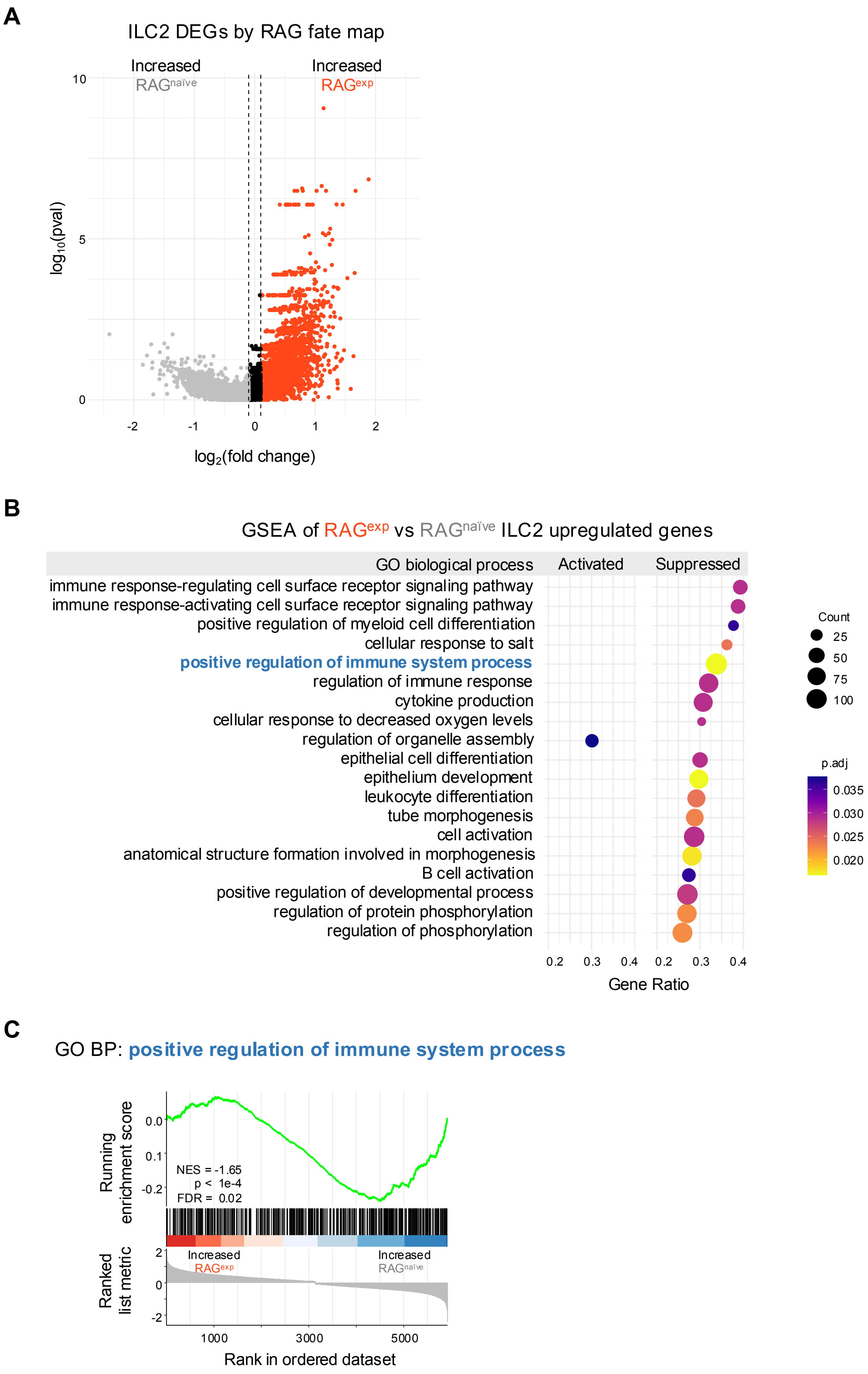
A
ILC2 DEGs by RAG fate map
Increased
RAGnaïve
Increased
RAGexp
log10(pval)
log2(fold change)
B
GSEA of RAGexp vs RAGnaïve ILC2 upregulated genes
GO biological process
Activated
Suppressed
immune response-regulating cell surface receptor signaling pathway
immune response-activating cell surface receptor signaling pathway
positive regulation of myeloid cell differentiation
cellular response to salt
positive regulation of immune system process
regulation of immune response
cytokine production
cellular response to decreased oxygen levels
regulation of organelle assembly
epithelial cell differentiation
epithelium development
leukocyte differentiation
tube morphogenesis
cell activation
anatomical structure formation involved in morphogenesis
B cell activation
positive regulation of developmental process
regulation of protein phosphorylation
regulation of phosphorylation
Gene Ratio
Count
p.adj
C
GO BP: positive regulation of immune system process
Running enrichment score
NES = -1.65
p < 1e-4
FDR = 0.02
Ranked list metric
Increased
RAGexp
Increased
RAGnaïve
Rank in ordered dataset

Figure S9

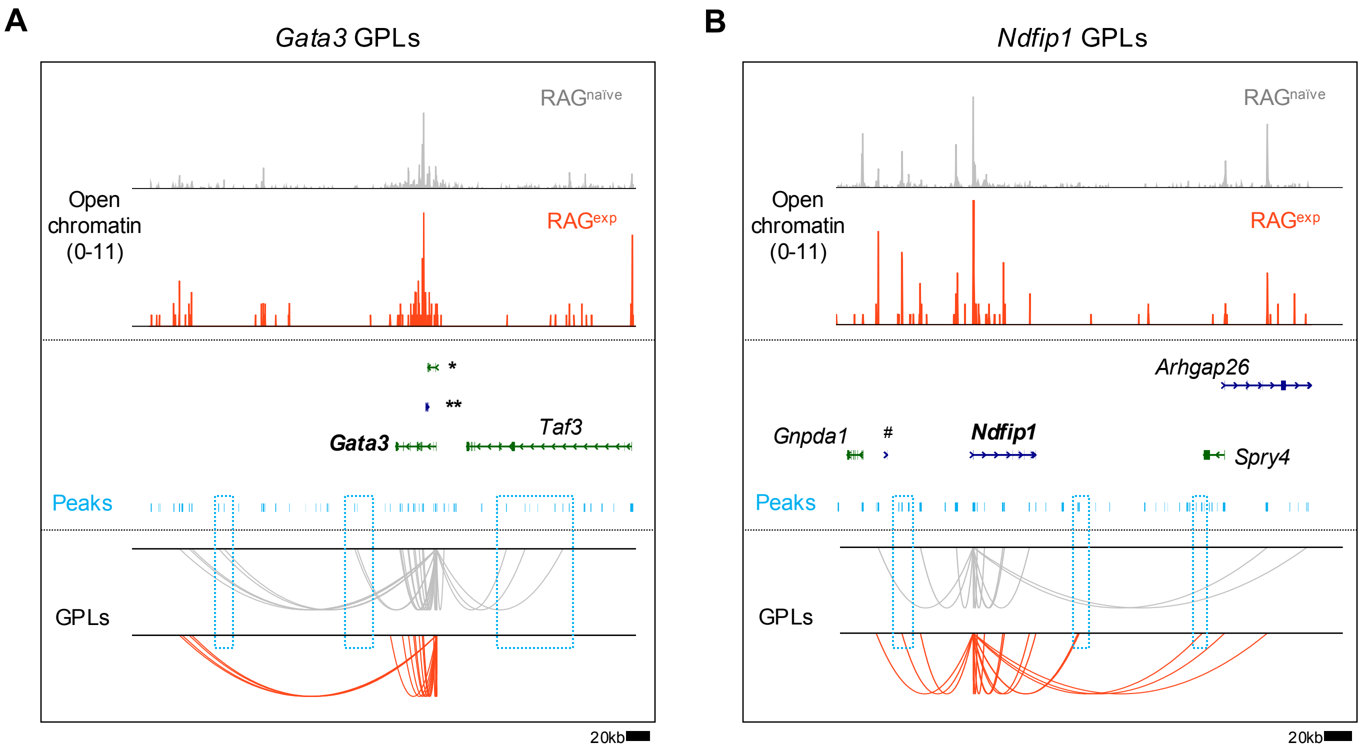
A
Gata3 GPLs
RAGnaïve
Open chromatin (0-11)
RAGexp
*
**
Gata3
Taf3
Peaks
GPLs
20kb
B
Ndfip1 GPLs
RAGnaïve
Open chromatin (0-11)
RAGexp
Arhgap26
Gnpda1
#
Ndfip1
Spry4
Peaks
GPLs
20kb

Figure S10

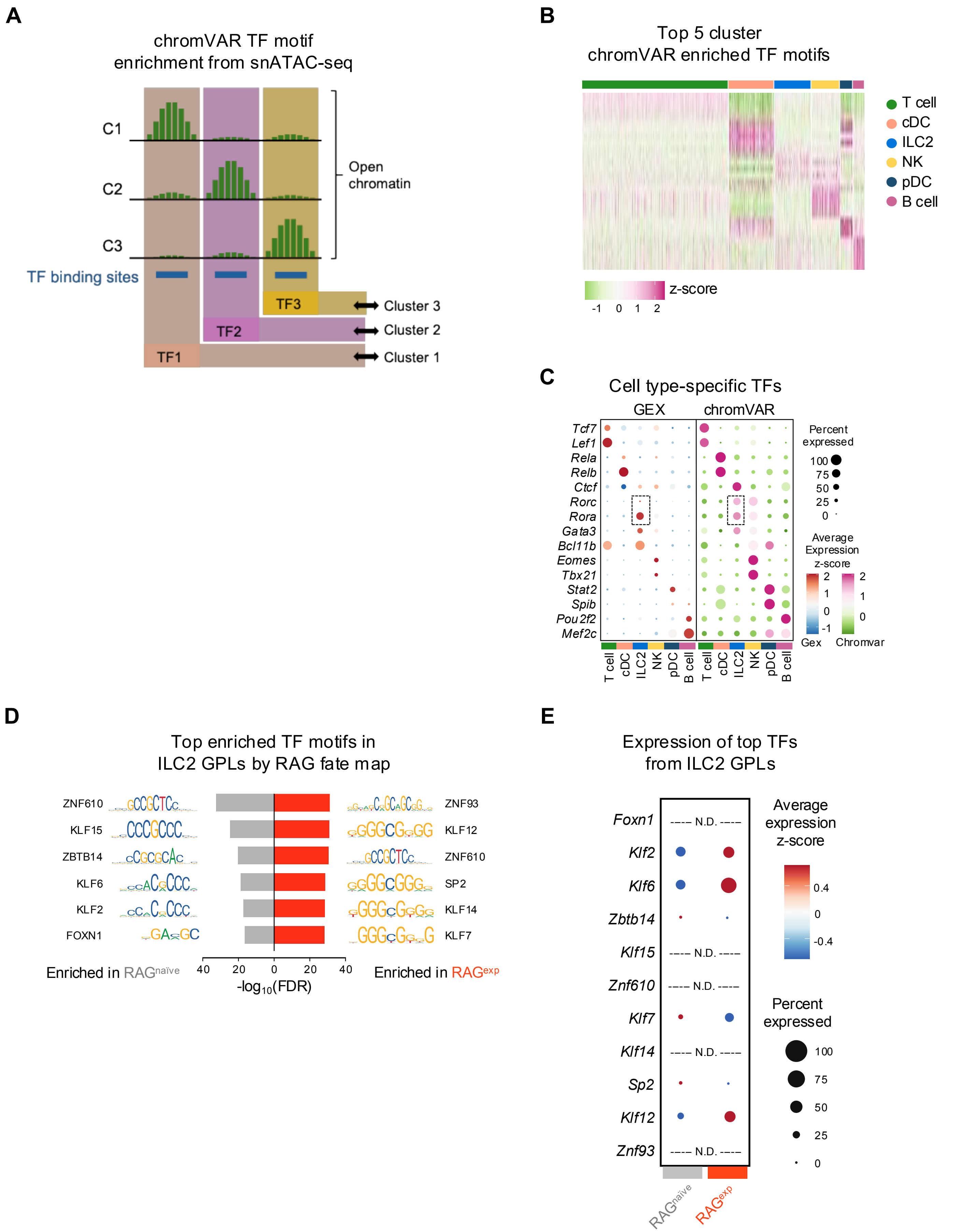
A
chromVAR TF motif
enrichment from snATAC-seq
C1
C2
C3
Open
chromatin
TF binding sites
TF3
TF2
TF1
Cluster 3
Cluster 2
Cluster 1
B
Top 5 cluster
chromVAR enriched TF motifs
T cell
cDC
ILC2
NK
pDC
B cell
z-score
-1 0 1 2
C
Cell type-specific TFs
GEX
chromVAR
Tcf7
Lef1
Rela
Relb
Ctcf
Rorc
Rora
Gata3
Bcl11b
Eomes
Tbx21
Stat2
Spib
Pou2f2
Mef2c
Percent
expressed
100
75
50
25
0
Average
Expression
z-score
Gex
Chromvar
T cell cDC ILC2 NK pDC B cell
D
Top enriched TF motifs in
ILC2 GPLs by RAG fate map
ZNF610
KLF15
ZBTB14
KLF6
KLF2
FOXN1
ZNF93
KLF12
ZNF610
SP2
KLF14
KLF7
Enriched in RAGnaïve
40 20 0 20 40
-log10(FDR)
Enriched in RAGexp
E
Expression of top TFs
from ILC2 GPLs
Foxn1
Klf2
Klf6
Zbtb14
Klf15
Znf610
Klf7
Klf14
Sp2
Klf12
Znf93
N.D.
Average
expression
z-score
0.4
0
-0.4
Percent
expressed
100
75
50
25
0
RAGnaïve
RAGexp

Figure S11

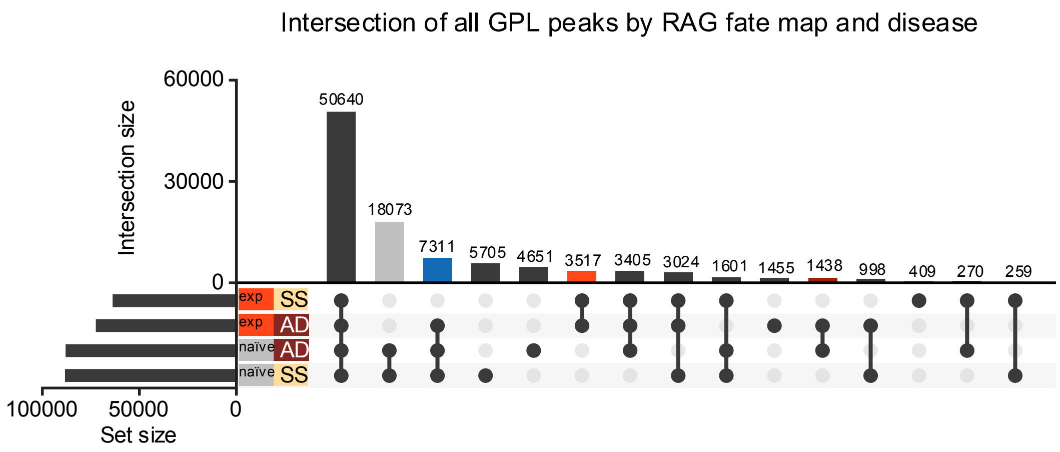

Intersection of all GPL peaks by RAG fate map and disease
Intersection size
60000
30000
0
50640
18073
7311
5705
4651
3517
3405
3024
1601
1455
1438
998
409
270
259
exp SS
exp AD
naïve AD
naïve SS
100000
50000
0
Set size

Figure S12

A

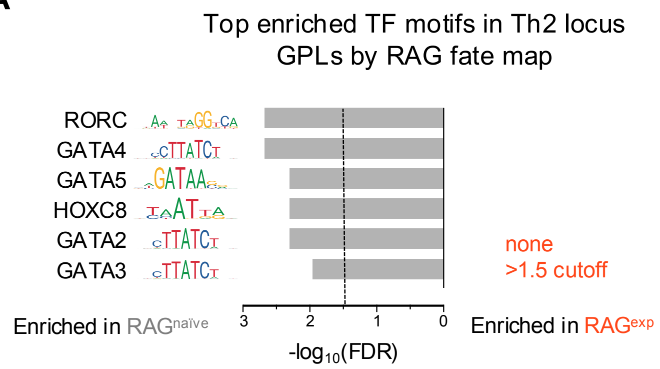

B

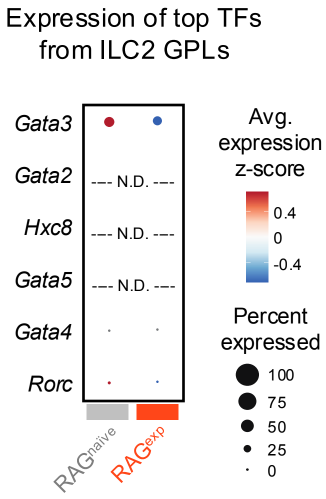

Supplement: Supplement 2 [file NIHPP2024.04.23.590767v2-supplement-2.pdf]
