# Supplementary material for: RAG suppresses group 2 innate lymphoid cells
Source: bioRxiv. 2024 Apr 28:2024.04.23.590767. Preprint. [Version 1] doi: 10.1101/2024.04.23.590767 (PMC11071423; doi:10.1101/2024.04.23.590767)
Supplement: Supplementary file 1 [file NIHPP2024.04.23.590767v1-supplement-1.pdf]

Figure S1

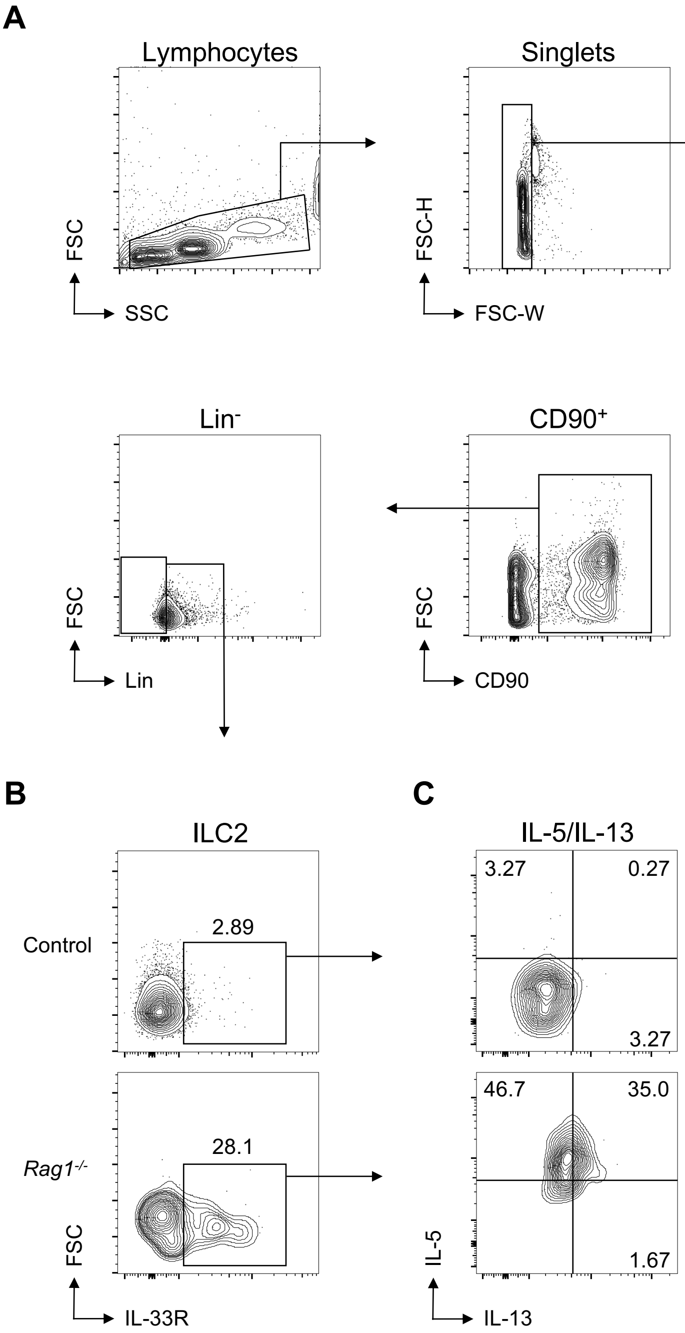
A
Lymphocytes
Singlets
Singlets
FSC
SSC
FSC-H
FSC-W
SSC-H
SSC-W
Lin-
CD90+
CD45+
FSC
Lin
FSC
CD90
FSC
CD45
B
C
ILC2
IL-5/IL-13
Control
2.89
3.27
0.27
3.27
Rag1-/-
28.1
46.7
35.0
1.67
FSC
IL-33R
IL-5
IL-13

Figure S2

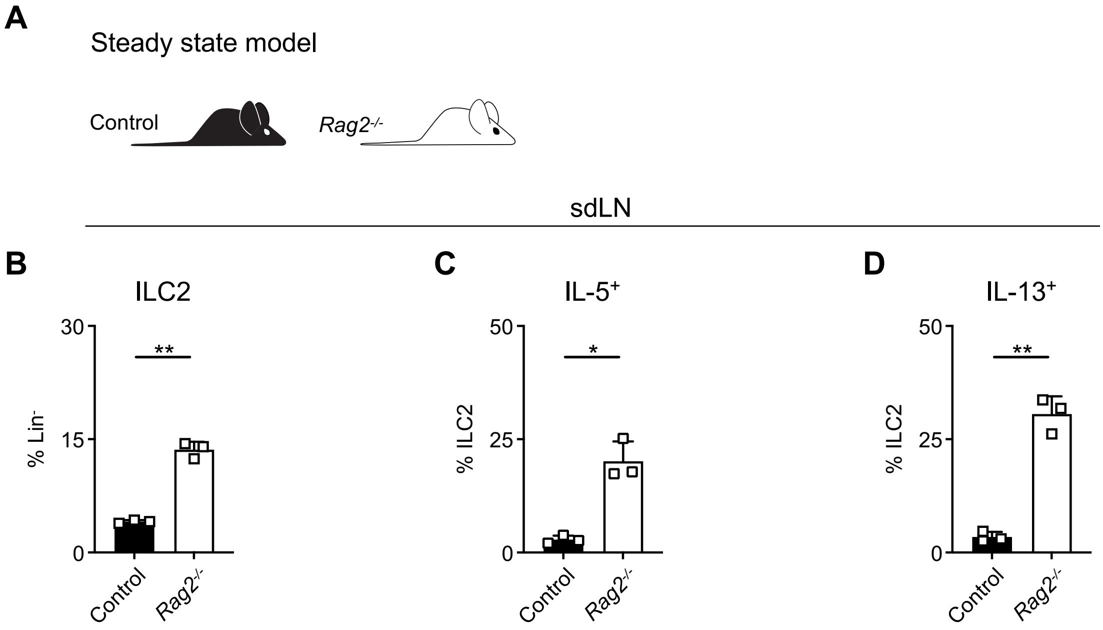
A
Steady state model
Control
Rag2-/-
sdLN
B
ILC2
% Lin-
30
15
0
**
Control
Rag2-/-
C
IL-5+
% ILC2
50
25
0
*
Control
Rag2-/-
D
IL-13+
% ILC2
50
25
0
**
Control
Rag2-/-

Figure S3

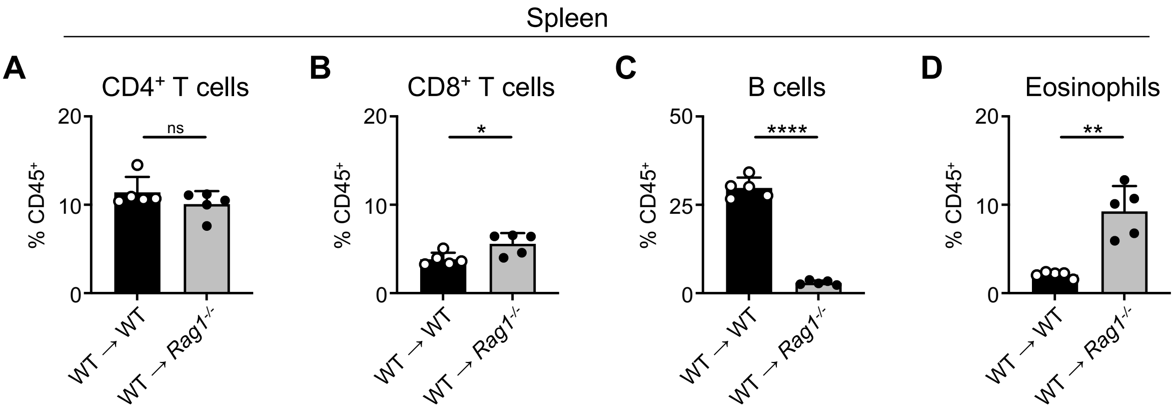

Spleen
A
CD4+ T cells
% CD45+
20
10
0
ns
WT → WT
WT → Rag1-/-
B
CD8+ T cells
% CD45+
20
10
0
*
WT → WT
WT → Rag1-/-
C
B cells
% CD45+
50
25
0
****
WT → WT
WT → Rag1-/-
D
Eosinophils
% CD45+
20
10
0
**
WT → WT
WT → Rag1-/-

Figure S4

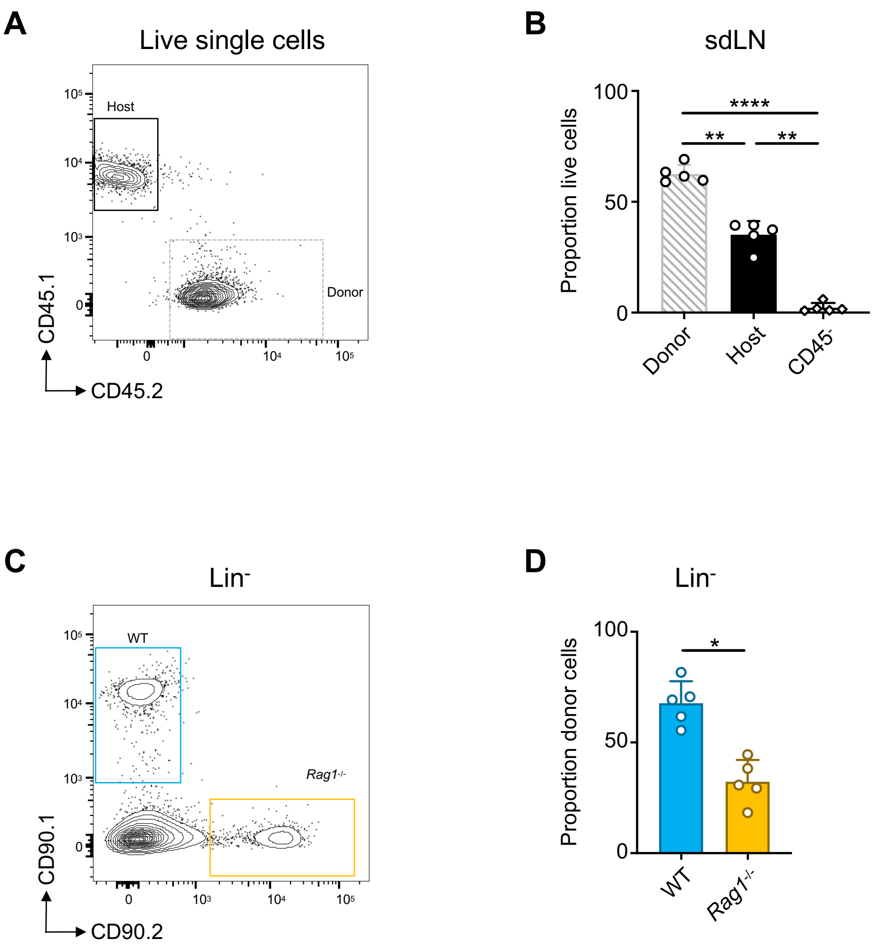
A
Live single cells
Host
Donor
CD45.1
CD45.2
B
sdLN
Proportion live cells
****
**
**
Donor
Host
CD45-
C
Lin-
WT
Rag1-/-
CD90.1
CD90.2
D
Lin-
Proportion donor cells
*
WT
Rag1-/-

Figure S5

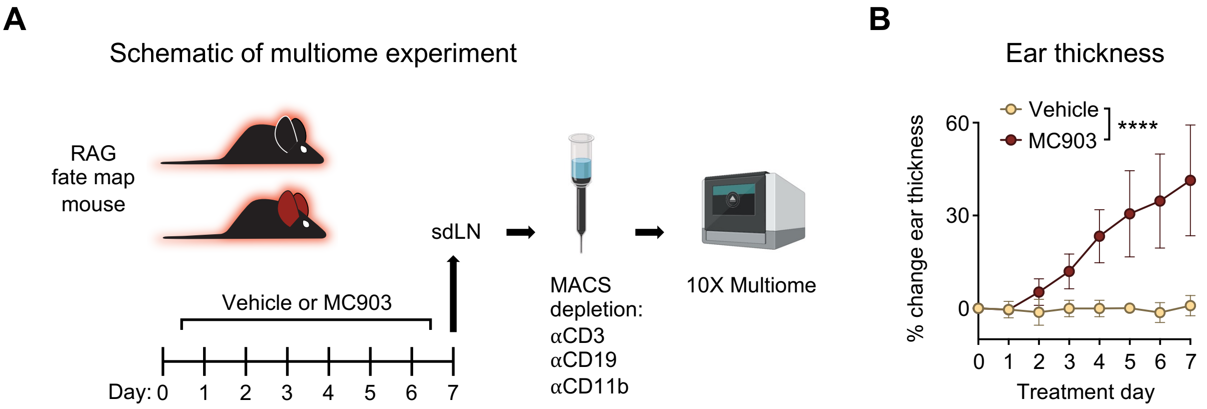
A
Schematic of multiome experiment
RAG
fate map
mouse
sdLN
MACS
depletion:
αCD3
αCD19
αCD11b
10X Multiome
Vehicle or MC903
Day: 0 1 2 3 4 5 6 7
B
Ear thickness
Vehicle
MC903
****
% change ear thickness
60
30
0
0 1 2 3 4 5 6 7
Treatment day

Figure S6

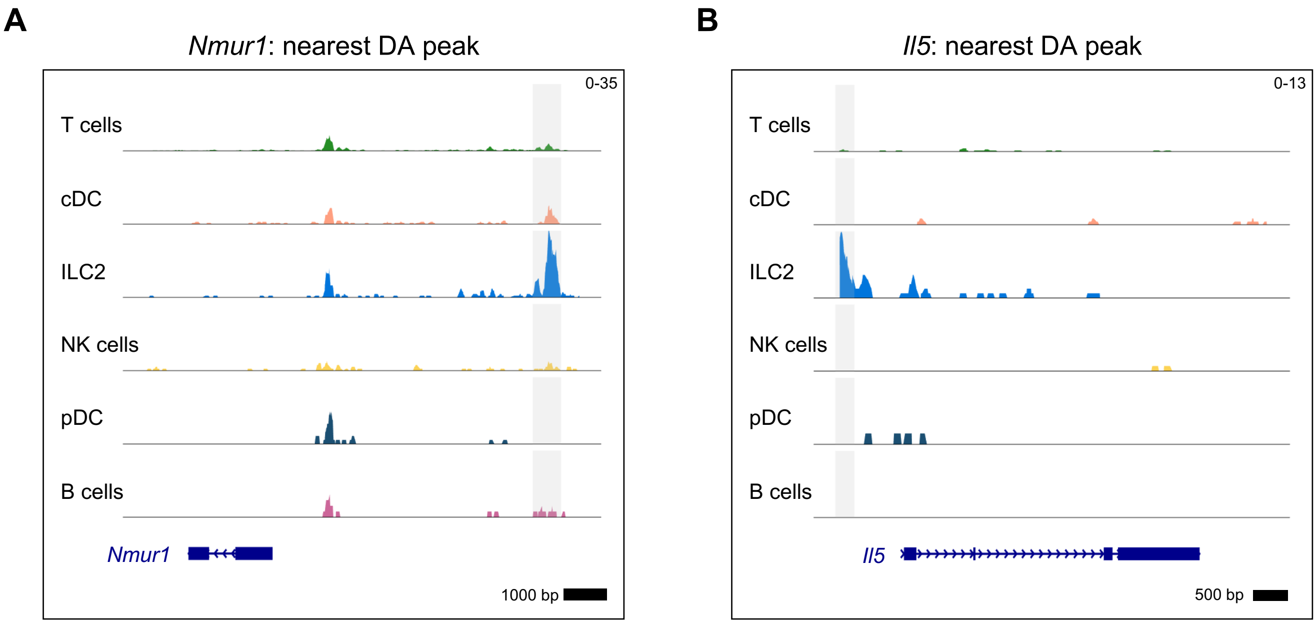
A
Nmur1: nearest DA peak
0-35
T cells
cDC
ILC2
NK cells
pDC
B cells
Nmur1
1000 bp
B
Il5: nearest DA peak
0-13
T cells
cDC
ILC2
NK cells
pDC
B cells
Il5
500 bp

Figure S7

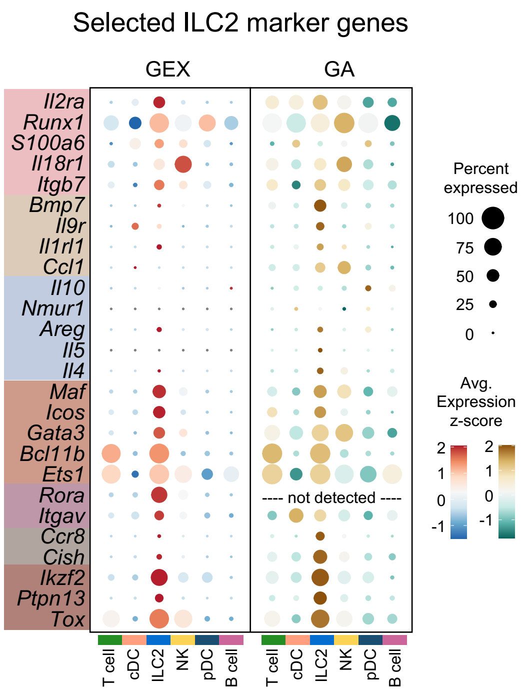
Selected ILC2 marker genes
GEX
GA
Il2ra
Runx1
S100a6
Il18r1
Itgb7
Bmp7
Il9r
Il1rl1
Ccl1
Il10
Nmur1
Areg
Il5
Il4
Maf
Icos
Gata3
Bcl11b
Ets1
Rora
Itgav
Ccr8
Cish
Ikzf2
Ptpn13
Tox
---- not detected ----
T cell
cDC
ILC2
NK
pDC
B cell
Percent expressed
100
75
50
25
0
Avg. Expression z-score
2
1
0
-1

Figure S8

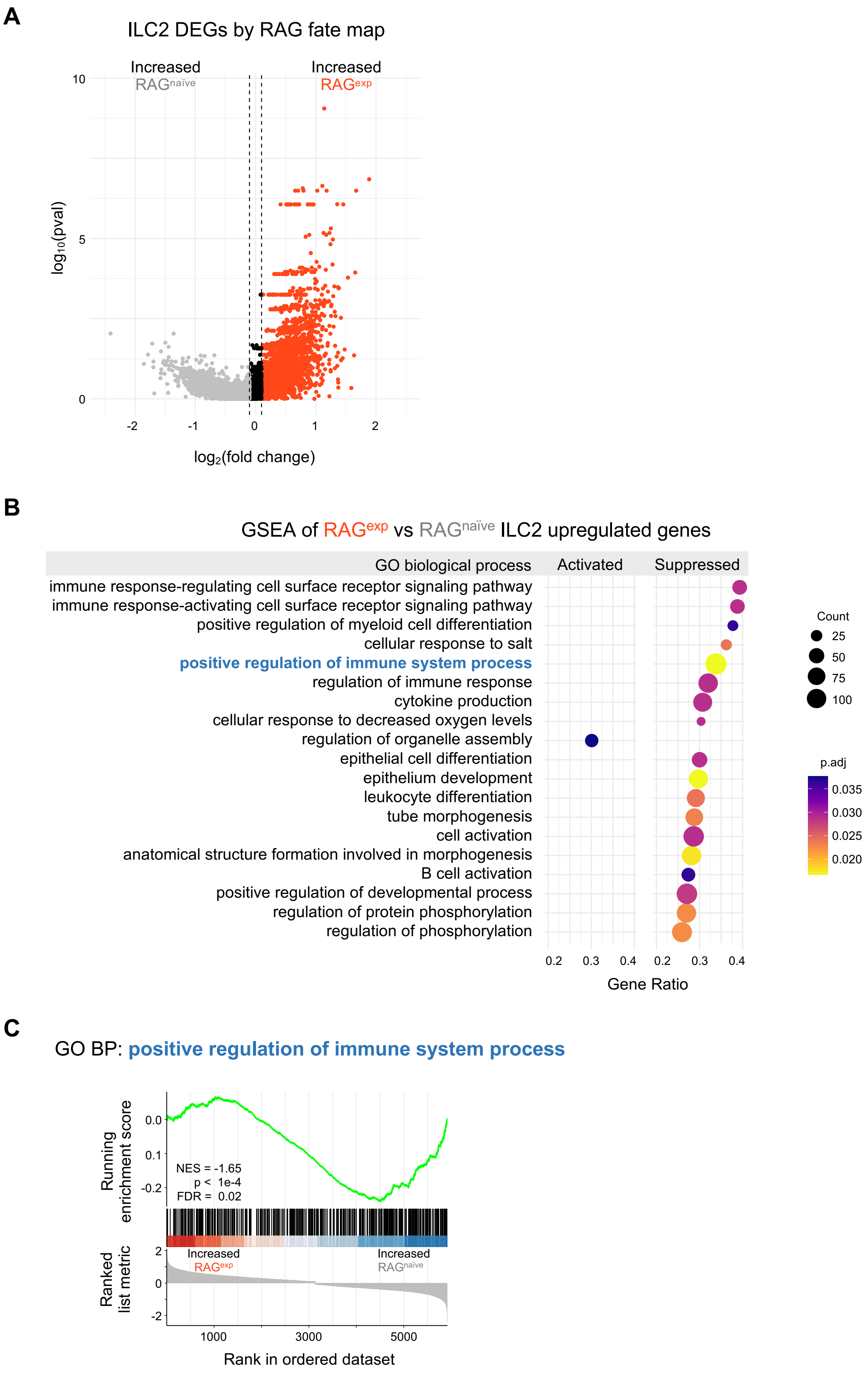
A
ILC2 DEGs by RAG fate map
Increased RAGnaïve
Increased RAGexp
log10(pval)
log2(fold change)
B
GSEA of RAGexp vs RAGnaïve ILC2 upregulated genes
GO biological process
Activated
Suppressed
immune response-regulating cell surface receptor signaling pathway
immune response-activating cell surface receptor signaling pathway
positive regulation of myeloid cell differentiation
cellular response to salt
positive regulation of immune system process
regulation of immune response
cytokine production
cellular response to decreased oxygen levels
regulation of organelle assembly
epithelial cell differentiation
epithelium development
leukocyte differentiation
tube morphogenesis
cell activation
anatomical structure formation involved in morphogenesis
B cell activation
positive regulation of developmental process
regulation of protein phosphorylation
regulation of phosphorylation
Gene Ratio
Count
25
50
75
100
p.adj
0.035
0.030
0.025
0.020
C
GO BP: positive regulation of immune system process
Running enrichment score
NES = -1.65
p < 1e-4
FDR = 0.02
Increased RAGexp
Increased RAGnaïve
Ranked list metric
Rank in ordered dataset

Figure S9

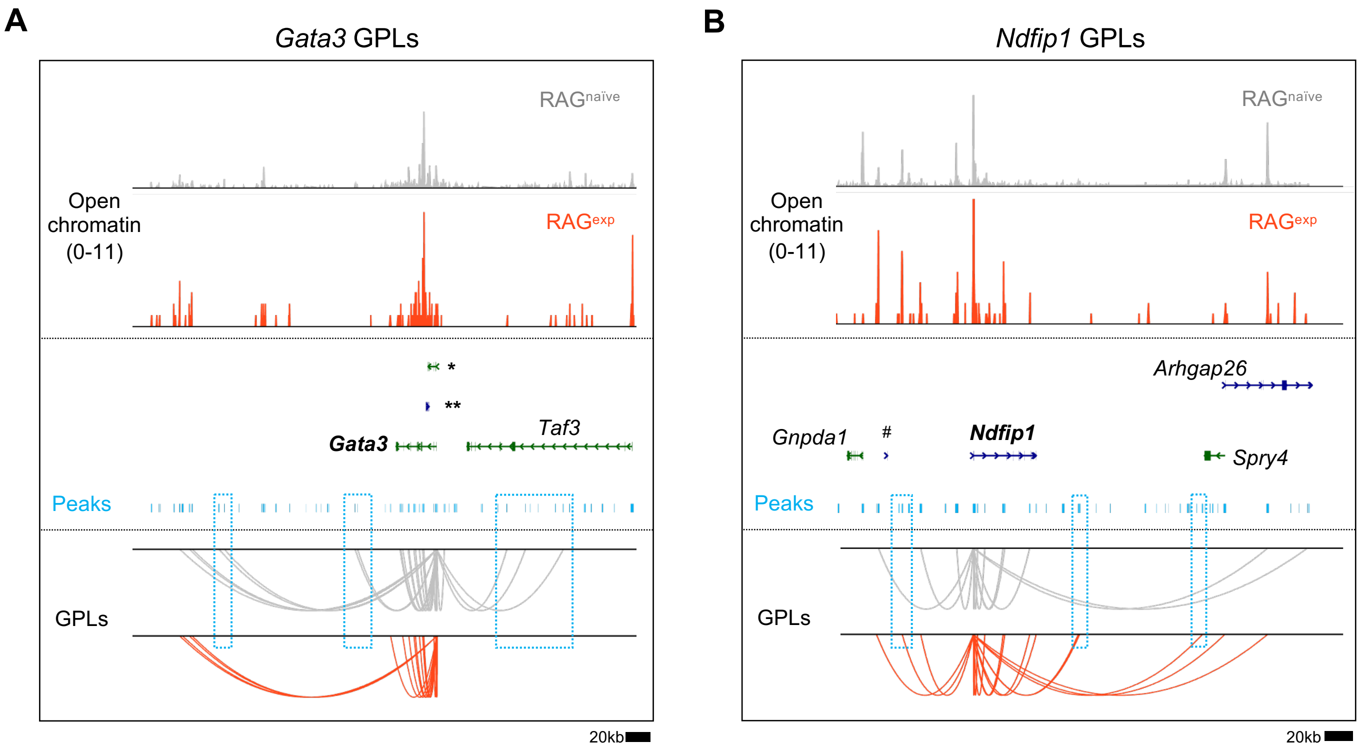
A
Gata3 GPLs
RAGnaïve
Open chromatin (0-11)
RAGexp
*
**
Gata3
Taf3
Peaks
GPLs
20kb
B
Ndfip1 GPLs
RAGnaïve
Open chromatin (0-11)
RAGexp
Arhgap26
Gnpda1
#
Ndfip1
Spry4
Peaks
GPLs
20kb

Figure S10

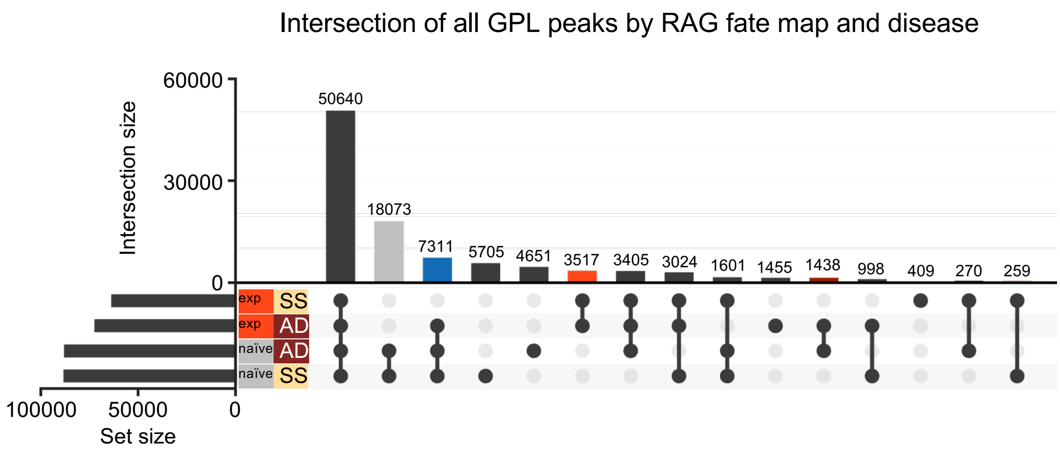
Intersection of all GPL peaks by RAG fate map and disease
60000
30000
0
Intersection size
50640
18073
7311
5705
4651
3517
3405
3024
1601
1455
1438
998
409
270
259
exp SS
exp AD
naïve AD
naïve SS
100000
50000
0
Set size
